# Detection of a Left Superior Vena Cava during a Pacemaker Implantation in Cotonou

**DOI:** 10.1155/2017/7634082

**Published:** 2017-08-08

**Authors:** A. Sonou, M. Hounkponou, L. Codjo, P. M. Adjagba, C. Houehanou, H. Dohou, S. Assani, Y. Tchabi, M. Houenassi

**Affiliations:** ^1^Department of Cardiology, Departmental Teaching Hospital of Ouémé-Plateau, University of Abomey-Calavi, Porto-Novo, Benin; ^2^Department of Cardiology, Teaching Hospital of Cotonou, University of Abomey-Calavi, Cotonou, Benin; ^3^Department of Cardiology, Departmental Teaching Hospital of Borgou-Alibori, University of Parakou, Parakou, Benin; ^4^Department of Cardiology, Army Hospital of Parakou, Parakou, Benin

## Abstract

Persistent left superior vena cava (LSVC) is a rare congenital anomaly. Its prevalence in the general population is 0.1 to 0.5%. LSVC is 5 times rarer when accompanied by an absence of the right superior vena cava (RSVC). We present the case of a 54-year-old man who carries a persistent LSVC without RSVC. Clinically, this patient presented a regular bradycardia at 40 per minute associated with a heart failure syndrome. The electrocardiogram diagnosed a complete atrioventricular block and transthoracic echocardiography showed dilated left heart cavities and a left ventricular ejection fraction of 50%. During the procedure of pacemaker implantation, the probe followed an unusual LSVC-coronary sinus-right atrium path and it was not easy to pass through the tricuspid orifice. We propose a review of the literature on this subject, focusing on the clinical implications of this malformation in cardiac stimulation and in other areas of cardiology.

## 1. Introduction

The persistence of LSVC was described for the first time in 1738 by Le Cat. It is the most common congenital anomaly of systemic venous return with a prevalence ranging from 0.1 to 0.5% in the general population [1]. It can be isolated or associated with other congenital heart diseases. Usually, it does not pose a haemodynamic problem. This abnormality can be encountered in various circumstances including cardiac surgery and any intervention including catheterization of the right cardiac cavities [2]. We report in this article a case of persistence of the LSVC detected during a pacemaker implantation. After a brief presentation of the clinical case, we will review the existing literature on the subject. Finally, we will talk about the lead's placing technique into the right ventricle during pacemaker implantation in this situation.

## 2. Case Report

We report the case of a 54-year-old man, a former smoker who complained of dyspnea and dizziness. Physical examination noted blood pressure at 170/80 mmHg and signs of heart failure and regular bradycardia at 40 per minute. The diagnosis of complete atrioventricular block with wide QRS complex (complete right bundle branch block) was made on the electrocardiogram. Transthoracic echocardiography showed moderately dilated left cardiac cavities (left ventricular end-diastolic diameter at 62 millimeters), high left ventricular filling pressure, and a dilated inferior vena cava. There was no pulmonary arterial hypertension.

**Figure 1:**
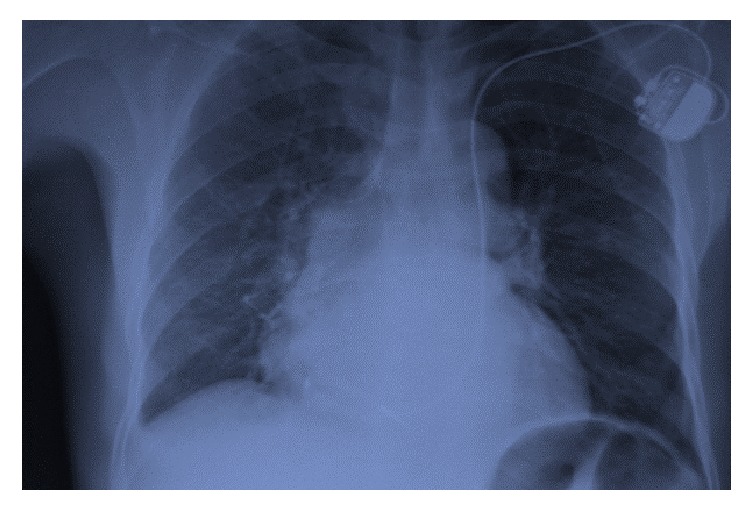
Chest X-ray seen from the front showing the cardiac stimulation lead descending into the LSVC, making a loop in the right atrium before being fixed in the right ventricle, just behind tricuspid valve.

We decided to implant a cardiac pacemaker and the patient was taken to the cardiac catheterization room. After its introduction into the left cephalic vein, the right ventricular pacing lead descended along the left side of the spine following the path of a LSVC and entered the right atrium through the coronary sinus (Figure 1). The attempt to ascend the stimulation lead into the RSVC starting from the right atrium failed, suggesting the persistence of LSVC with absence of RSVC.

Once in the right atrium, it was necessary to make a loop with the lead in order to reach the tricuspid orifice, remove the curved guide, replace it by a straight guide, pass through the tricuspid valve, and screw the lead into the right ventricle. A good stimulation threshold of 0.4 volts and a sensitivity of 12 millivolts were obtained intraoperatively and were maintained over a minimum period of 6 months after implantation. Clinically, the patient showed no signs of heart failure and hemodynamics remained stable during successive checks.

## 3. Discussion

Development of the superior vena cava system begins with the sinus venosus, the main venous formation in which 3 pairs of cardinal, umbilical, and vitelline veins are discharged. The right cardinal vein is transformed into RSVC, the left cardinal vein into LSVC, and the left part of the sinus venosus into the coronary sinus. In normal embryogenesis, there is progressive involution and disappearance of the left cardinal vein by extrinsic compression of the left atrium and the left hilum. An incomplete regression of the left cardinal vein will lead to the persistence of the LSVC [3]. Its prevalence ranges from 0.5% in the general population to 5% in congenital cardiopathy carriers and 4% in patients undergoing electrophysiology for a rhythm disorder [4]. In subjects with LSVC, RSVC is frequently present in 80% of cases [5]. Its absence means that all blood of superior system vena cava (2 subclavian veins and the 2 internal jugular veins) flows into LSVC and a coronary sinus often dilated. This situation has rarely been reported with a prevalence ranging from 0.07 to 0.13% of patients with congenital or acquired heart disease [6]. In the Dan Adams study about 26 LSVC carriers operated for congenital heart disease between 1968 and 1973, LSVC had entered the right atrium in 77.3% of cases compared to 22.7% flowed into left atrium [7]. The implications of LSVC's persistence will be discussed in the context of pacemaker implantation and in others areas of cardiology.

### 3.1. Apart from Cardiac Stimulation

Although this venous return abnormality has important clinical implications, it has no particular symptom and is hemodynamically well tolerated. LSVC's connection with left atrium produces a right-left shunt with severe cyanosis. LSVC can prevent the placement of a central catheter in patients with cancer as well as cardiac catheterization to measure right cardiac chambers' pressures. Realization of a mediastinal surgery without being aware of LSVC's existence can lead to dangerous hemorrhagic complications [8]. LSVC has been incriminated in the genesis of atrial fibrillation but also in atrioventricular conduction disorders [9].

### 3.2. During Pacemaker's Implantation

Presence of LSVC makes the implantation of a cardiac pacing lead into the right ventricle particularly difficult. The explanation for this difficulty is that the ostium of the coronary sinus is not aligned with the tricuspid orifice and that a loop must be made before a lead can pass the tricuspid valve. Many techniques have been proposed to facilitate this passage [10–13].

The first technique has been summarized in 4 phases with a total duration of 1 to 4 minutes:

- Advance the lead with a straight guide until it crosses the coronary sinus and enters the right atrium.
- Replace the straight guide with a preformed J-guide (conventionally used for the right atrial lead) and position the lead against the lateral or anterolateral wall of the right atrium.
- Remove the guide, 3–5 cm.
- Then push the probe which forms a loop and passes easily the tricuspid orifice [3].

This technique has given excellent results in literature in terms of passage of the lead through the tricuspid valve with good stimulation and detection's parameters [14, 15] without making it necessary to restart the procedure on the opposite side. A resumption of the procedure on the right would be a failure if there was as in the clinical case absence of the RSVC.

A second technique involves the use of a 9 F sheath which can be guided to the tricuspid orifice and allow the lead to be deployed [14]. The first technique has been used in our case. We believe that a sheath is more traumatic than a lead and irritation of the coronary sinus could cause arterial hypotension, angina pain, or even cardiac arrest [2]. A dilated right atrium may increase the difficulty of passing a lead through the tricuspid valve regardless of the method used. However, LSVC facilitates the implantation of a left ventricular lead during the placement of a triple chamber pacemaker. Persistent LSVC is rare and generally discovered in adult cardiology during a pacemaker implantation. However, LSVC may be strongly suspected in transthoracic echocardiography in presence of an abnormally dilated coronary sinus [16]. The subsequent injection into a left antecubital vein of an emulsion, made by saline serum or glucose serum, will successively lead to opacification of the dilated coronary sinus and the right atrium. In contrast, an injection of emulsion in a right antecubital vein would have opacified the right cardiac cavities without previously opacifying the coronary sinus [17, 18]. This simple, noninvasive procedure is easy to apply because it just requires an echocardiograph.

## 4. Conclusion

We reported a clinical case of persistent LSVC with absence of RSVC. This malformation has implications in cardiologic practice during a pacemaker implantation and in other areas of cardiology. Different techniques can be used to facilitate the passage through the tricuspid valve of a cardiac stimulation lead in this situation. Transthoracic echocardiography can detect this malformation prior to any procedure with which its existence may interfere.
